# *In vitro* study of antifungal effects of earthworm coelomic fluid obtained from *Eisenia fetida* on three opportunistic fungal pathogens

**DOI:** 10.22034/cmm.2025.345248.1637

**Published:** 2025-06-30

**Authors:** Saeed Sanaiirad, Mehrdad Seifi, Negar Hemmati, Alireza Khosravi, Donya Nikaein

**Affiliations:** Department of Microbiology and Immunology, Faculty of Veterinary Medicine, University of Tehran, Tehran, Iran

**Keywords:** Antifungal, *Aspergillus fumigatus*, *Candida albicans*, Earthworm, *Eisenia Fetida*

## Abstract

**Background and Purpose:**

Pathogenic fungi, including both true and opportunistic pathogens, pose significant health risks, particularly in immunocompromised individuals. Species, such as *Candida albicans*, *Aspergillus fumigatus*, and *Cryptococcus neoformans*, cause fatal infections and frequently develop resistance to conventional antifungal therapies. Limitations of current antifungal medications, such as drug toxicity, resistance development, and environmental concerns, highlight the urgent need for novel therapeutic strategies. Earthworm extracts, particularly those derived from *Eisenia fetida*, have been recognized as a promising alternative in traditional Chinese medicine. This study aimed to assess the antifungal effects of a peptide extract from *E. fetida* against these opportunistic fungal pathogens.

**Materials and Methods:**

The earthworm extract was obtained from *E. fetida* through electroporation and centrifugation to isolate bioactive components. Composition of the extract was analyzed in detail; accordingly, protein content was determined using the Bradford and Kjeldahl methods, fat content was measured via Soxhlet extraction, and moisture, dry matter, and ash contents were also quantified to provide a comprehensive profile. To evaluate antifungal activity, fungal cultures of *A. fumigatus*, *C. albicans*, and *C. neoformans* were grown on Sabouraud dextrose agar. The disk diffusion method was used to assess antifungal activity by measuring inhibition zones surrounding extract-containing disks. A dilution series of the *E. fetida* extract was also prepared to further analyze antifungal effects. The broth microdilution method was employed to determine the minimum inhibitory concentration (MIC) and minimum fungicidal concentration (MFC) for each fungal species, providing quantitative data on the effectiveness of the extract.

**Results:**

The coelomic fluid extracted from *E. fetida* contained 60.03% protein, 8.136% fat, 6.91% ash, 6.03% moisture, and 8.65% dry matter. Disk diffusion assays revealed significant inhibition of *A. fumigatus* and *C. albicans*, with the extract exhibiting stronger effects at higher concentrations. In broth microdilution assays, the extract achieved MIC/MFC values of 12.5%/25% against *A. fumigatus* and 3.125%/6.25% against *Candida albicans*. However, its efficacy against *C. neoformans* was lower, while the commercial antifungal drug, itraconazole, demonstrated superior efficacy against all tested strains.

**Conclusion:**

Earthworm extracts, rich in antimicrobial peptides, exhibit promising antifungal properties, particularly against *C. albicans*. Although not as effective as itraconazole, the potential of the extract as a safer and environmentally friendly alternative underscores its significance in antifungal research. Further studies are needed to enhance its efficacy and broaden its antifungal spectrum, potentially leading to new, sustainable strategies for managing fungal infections.

## Introduction

Fungal infections have emerged as a growing global health concern, accounting for approximately 1.5 million deaths each year [ 1 , 2 ]. These infections are generally caused by two types of fungal pathogens: true pathogens, capable of infecting healthy individuals, and opportunistic pathogens, which primarily affect immunocompromised individuals, such as cancer patients undergoing chemotherapy, individuals with human immunodeficiency virus/acquired immunodeficiency syndrome, and organ transplant recipients [ 3 - 6 ]. Among the most clinically significant species are *Candida albicans*, *Aspergillus fumigatus*, and *Cryptococcus neoformans*, all of which can cause severe and often drug-resistant infections [ 7 , 8 ].

*Candida albicans* is a common commensal organism found in the gastrointestinal tract, oral cavity, and vaginal mucosa [ 9 , 10 ]. However, under conditions of immunosuppression or microbiota imbalance, it can overgrow and cause candidiasis, presenting in forms ranging from superficial infections, like oral thrush, to life-threatening systemic disease [ 11 - 13 ]. The increasing incidence of azole-resistant *Candida* strains highlights the pressing need for new antifungal therapies [ 14 , 15 ].

*Aspergillus fumigatus* is a filamentous fungus commonly found in soil and decaying organic matter [ 16 ]. It is responsible for aspergillosis, which may manifest as allergic reactions, chronic pulmonary conditions, or invasive disease, especially in immunocompromised individuals [ 17 - 19 ]. Resistance to standard antifungal treatments has made managing invasive aspergillosis particularly challenging [ 20 ]. *Candida neoformans* is a yeast-like fungus that causes cryptococcosis, often presenting as pulmonary or central nervous system infections in immunocompromised hosts [ 20 - 22 ]. Its polysaccharide capsule is a key virulence factor [ 23 ]. Current treatments, primarily amphotericin B and flucytosine, are limited by toxicity and emerging resistance, underscoring the need for safer and more effective alternatives [ 24 ].

Current limitations in antifungal therapy, including drug toxicity, resistance, adverse side effects, high treatment costs, and ecological concerns, highlight the necessity of offering more effective therapeutic options [ 25 , 26 ]. Consequently, developing innovative strategies for preventing and treating fungal infections in humans, animals, and plants is essential. Natural products have long served as a rich source of pharmacologically active compounds, with many modern drugs originating from traditional medicine [ 27 , 28 ]. In traditional Chinese medicine, invertebrates, especially earthworms, have been widely used for their potential therapeutic benefits [ 29 - 31 ]. Despite their long-standing use, the antifungal potential of earthworms remains unexplored, mainly in scientific research, particularly against pathogens that affect humans and animals [ 29 , 32 , 33 ]. *Eisenia fetida*, a widely studied earthworm species, is crucial in maintaining soil health [ 34 , 35 ]. This species possesses unique physiological traits, including the circulation of coelomic fluid and a strong innate immune system, which may contribute to its resilience to infections [ 36 , 37 ].

Previous studies have demonstrated the antimicrobial properties of earthworm-derived compounds, particularly in the coelomic fluid of *E. fetida* [ 38 ]. Research has shown that these bioactive compounds possess significant antibacterial, antifungal, and antiviral activities [ 39 ]. The antimicrobial effects are thought to arise from a variety of bioactive molecules, such as proteins, peptides, and enzymes, which can disrupt microbial cell membranes, inhibit protein synthesis, and interfere with key metabolic pathways [ 40 ]. This study aimed to investigate the *in vitro* antifungal activity of coelomic fluid from *E. fetida* against *C. albicans*, *A. fumigatus*, and *C. neoformans*, aiming to evaluate its potential as a novel antifungal agent.

## Materials and Methods

### Preparing earthworm extract

To prepare the earthworm extract, multiple samples of *E. fetida* were obtained from the mycology laboratory at the Faculty of Veterinary Medicine, University of Tehran, Tehran, Iran. The earthworms were thoroughly cleaned to eliminate any adhering soil and particulate matter. This was achieved by submerging the worms in ultra-pure water for a period of 30 min , followed by gentle agitation to ensure complete removal of contaminants. After cleaning, the earthworms were allowed to dry on sterile filter paper to remove excess moisture. Subsequently, they were placed in a 10 × 35 mm Petri dish containing 500 μL of 0.1% sodium chloride solution in ultra-pure water. To facilitate the extraction of bioactive compounds, electroporation was performed by applying a voltage using a 9-volt battery [ 41 ]. The worms were subjected to ten brief pulses of voltage, each lasting less than one second, to enhance cell membrane permeability and promote the release of intracellular components, a method known as electroporation.

The resulting liquid extract was carefully transferred to a 1.5-ml Eppendorf tube. To maximize the yield of the extract, the Petri dish was rinsed with an additional 500 μL of the sodium chloride solution, and this rinse was also added to the Eppendorf tube. The mixture was then centrifuged at 10,000 rpm for 10 min at 4 °C to separate the solid debris from the liquid extract (Fanavaran, Iran). The supernatant was collected, and the liquid was evaporated overnight at room temperature to concentrate the extract. The dried extract was subsequently stored at -80 °C to preserve the integrity of the bioactive compounds for further antifungal activity assay [ 42 , 43 ].

### Protein content determination of Eisenia fetida extract

Protein concentration in *E. fetida* extract was measured using both the Bradford and Kjeldahl methods. The Bradford assay was conducted due to its sensitivity and simplicity. A spectrophotometer was calibrated at 595 nm using a blank solution (950 µL Bradford reagent + 50 µL distilled water) (Merck, Germany). A standard curve was generated using bovine serum albumin (Sigma-Aldrich, USA) at concentrations within the range of 0–2.0 mg/mL. For the test sample, 50 µL of the extract was mixed with 950 µL of Bradford reagent, vortexed, incubated for 5 min at room temperature, and measured at 595 nm. The absorbance values were plotted against the standard curve to determine protein concentration. All samples were analyzed in triplicate [ 44 - 46 ].

The Kjeldahl method was also employed to assess total nitrogen as a proxy for protein. Between 0.7 and 5.3 g of extract was digested with 7 g sodium sulfate, 1 g copper sulfate, and 20 mL concentrated sulfuric acid. Digestion was continued until a clear green solution indicated a complete breakdown of organic matter. After cooling, 300 mL of distilled water was added, and the mixture was distilled. Released ammonia was captured in a boric acid solution and titrated with 0.1 N HCl until the endpoint (color change from yellow to pink). Volume of acid used was recorded and used to calculate protein content using standard Kjeldahl formulas: $\mathrm{protein content (\%)}=\frac{\mathrm{(𝑉 𝐻𝐶𝐿 - 𝑉 𝑏𝑙𝑎𝑛𝑘)\times 𝑁 𝐻𝐶𝐿 \times 14.01 \times 6.25 \times 100}}{\mathrm{𝑆𝑎𝑚𝑝𝑙𝑒 𝑤𝑒𝑖𝑔ℎ𝑡 (𝑔𝑟)}}$ where V_HCL_ is the volume of hydrochloric acid used for titration (mL), V_blank_ is volume of hydrochloric acid used in the blank titration (mL), N_HCL_ is normality of hydrochloric acid (N), 14.01 is molecular weight of nitrogen (gr/mol), and 6.25 is conversion factor from nitrogen to protein [ 47 ].

### Measurement of fat content in Eisenia fetida extract by the Soxhlet method

The Soxhlet extraction method measures fat content by continuously extracting fat from a sample using a solvent. In this study, a fully automatic Soxhlet device was used to enhance accuracy and minimize potential errors [ 48 ].

### Measurement of moisture and dry matter of Eisenia fetida extract

Moisture and dry matter content of *E. fetida* extract were measured by gravimetric analysis. A clean, dry glass plate was heated at 135 °C for 20 min, cooled in a desiccator, and weighed (W_1_). Afterward, 10 g of sample was added, and the plate was reweighed (W_2_). The sample was dried at 135 °C for 5 h, cooled, and weighed again (W_3_). Moisture content was calculated using the following formula: $\mathrm{moisture content (\%)}=\frac{\mathrm{(w2 - w3) \times 100}}{\mathrm{(w2 - w1)}}$ = Where W_2_ is the weight of the plate with the sample before drying, W_3_ is the weight of the plate with the dried sample, and W_1_ is the weight of the empty plate [ 49 ]. The dry matter percentage was then determined through this formula: dry matter (%)=100 − moisture content (%) = .

### Measurement of ash content in Eisenia fetida extract samples

Ash content was determined by incinerating 2 g of the sample in a preheated crucible at 550 °C for 3-5 h until the residue turned white. The crucible was cooled in a desiccator and weighed (W_ash_). Ash content was calculated based on the following formula: $ash content (\%)=\frac{\mathrm{(𝑊𝑎𝑠ℎ- 𝑊𝑐𝑟𝑢𝑐𝑖𝑏𝑙𝑒) \times 100}}{\mathrm{𝑆𝑎𝑚𝑝𝑙𝑒 𝑊𝑒𝑖𝑔ℎ𝑡}}$ = Where W_ash_ is the weight of the crucible with ash, W_crucible_ is the weight of the empty crucible, and the sample weight is 2 g [ 50 ].

### Fungal culture and antifungal susceptibility testing

Three standard fungal strains were used in this study, namely *A. fumigatus* ATCC 90960 (a filamentous fungus), *C. albicans* ATCC 10231, and *C. neoformans* ATCC 90112 (both yeasts). These strains were obtained from the Mycology Laboratory of the Faculty of Veterinary Medicine, University of Tehran [ 51 ].

### Preparation of culture media

Sabouraud Dextrose Agar (SDA; Merck, Germany) supplemented with chloramphenicol (80 μg/mL; Sigma-Aldrich, USA) was prepared by dissolving the appropriate amount of SDA powder in distilled water, autoclaving at 121 °C for 20 min, and adding chloramphenicol after cooling to ~50 °C. The media were poured into sterile Petri dishes (Life Sciences, South Korea) and allowed to solidify [ 52 ].

### Inoculation and Incubation

For inoculation, each fungal strain was streaked onto the SDA plates using a sterile loop (Inoculating loop, HiMedia, India) under aseptic conditions. Plates were then sealed with parafilm (Parafilm M, Bemis, USA) to prevent contamination and incubated at 37 °C for optimal growth. The incubation period lasted for 24-48 h for yeasts (*C. albicans* and *C. neoformans*) and up to 14 days for *A. fumigatus*, which required a longer time to allow for proper colony formation. The temperature was maintained at 37 °C throughout the incubation process to simulate optimal growth conditions for the fungal species under study.

### Preparation of fungal suspensions

For yeasts (*C. albicans* and *C. neoformans*), a few colonies were transferred to phosphate-buffered saline (Gibco, USA) using a sterile loop and vortexed (IKA, Germany) to create a homogeneous suspension. For *A. fumigatus*, 5 mL of PST3 solution (0.1% Tween 80 [Merck, Germany] in physiological saline [Darou Pakhsh, Iran]) was added to the culture plate, and the conidia were dislodged with a sterile loop. The suspension was vortexed and allowed to settle at room temperature for 5-10 min. The upper conidial suspension was collected and counted using a Neubauer hemocytometer (Marienfeld, Germany)

All suspensions were adjusted to a final concentration of approximately 1 × 10^6^ CFU/mL for disk diffusion and 0.4 × 10^4^ to 5 × 10^4^ CFU/mL for microdilution, according to Clinical and Laboratory Standards Institute (CLSI) standards. Each Antifungal experiment was conducted independently in triplicate, and data are presented as the mean value with the corresponding standard deviation (mean ± SD).

### Disk diffusion assay

To evaluate the antifungal activity of the *E. fetida* peptide extract, the disk diffusion method was employed. This assay followed the guidelines of CLSI M44-A for yeasts (*C. albicans* and *C. neoformans*) and CLSI M51-A for *A. fumigatus*. Mueller–Hinton agar (HiMedia, India) supplemented with 2% glucose (Sigma-Aldrich, USA) was prepared and poured into 8 cm Petri dishes (Life Sciences, South Korea). The plates were used within 24 h of preparation to ensure the freshness of the medium. A 100 μL aliquot of each fungal suspension was evenly spread across the surface of the agar plate. After allowing the inoculum to dry, sterile blank disks (Oxoid, UK) were impregnated with 20 μL of the *E. fetida* peptide extract at concentrations of 100%, 50%, 25%, and 12.5% mg/mL, and placed on the surface of the agar. Commercial itraconazole disks (10 μg; Neosensit, Iran) were used as positive controls. The plates were then incubated at 35 °Cfor 24-48 h to allow for fungal growth and the development of inhibition zones. The zones of inhibition were measured in millimeters to evaluate the antifungal activity of the extract against the fungal strains [ 51 , 52 ].

### Broth microdilution assay (minimum inhibitory concentration testing)

The minimum inhibitory concentrations (MICs) were determined according to CLSI M38-A2 for *A. fumigatus* and CLSI M60 for the yeasts. The RPMI 1640 medium with 2% glucose (Gibco, USA) was used in 96-well microtiter plates (Nunc, Thermo Fisher Scientific, Denmark). Serial dilutions of itraconazole were prepared in RPMI1640 medium with 2% glucose (Gibco, USA), within the range of 0.21-100% concentration (0.21-100 mg/mL). These dilutions were added to wells 1 to 10 of the 96-well microplates, ensuring the appropriate final concentrations for the antifungal activity test. Fungal suspensions were added to each well (final volume: 200 µL). Plates were incubated at 30 °C for 24 h, and growth inhibition was assessed both visually and spectrophotometrically at 540 nm using an ELISA reader (BioTek Instruments, USA). The lowest concentration showing no visible growth was recorded as the MIC [ 53 ].

### Minimum fungicidal concentration

Aliquots (100 µL) from MIC wells and higher concentrations were plated onto SDA (Merck, Germany) and incubated for 14 days. The lowest concentration that yielded fewer than three colonies (99% inhibition) was recorded as the minimum fungicidal concentration (MFC) [ 53 , 54 ].

### Minimum fungicidal concentration/minimum inhibitory concentration ratio

The MFC/MIC ratio was calculated for each strain to determine whether the extract had fungistatic (ratio > 4) or fungicidal (ratio ≤ 4) activity [ 54 ].

## Results

**Figure 1:**
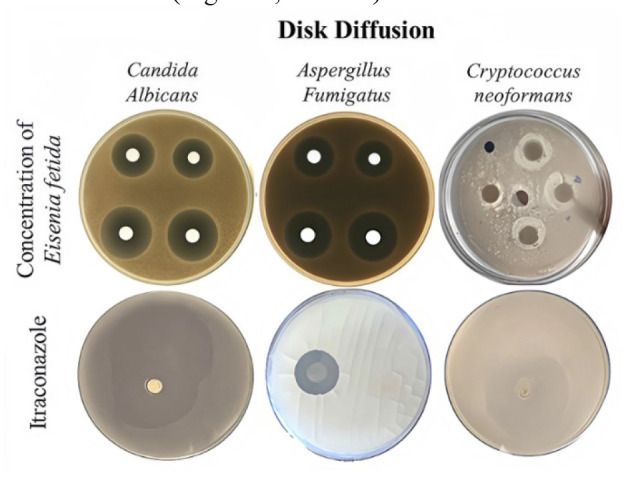
Results of pathogenic fungal growth inhibition in two laboratory methods

The results of the Disc Diffusion assay showed that the *E. fetida* extract exhibited varying degrees of inhibitory activity against *A .fumigatus*, *C. albicans*, and *C .neoformans*. For *A. fumigatus*, the zone of inhibition increased with higher concentrations of the extract, ranging from 1 cm at 12.5 μg to 1.7 cm at 100 μg. Similarly, for *C. albicans*, the inhibition zones ranged from 2.2 cm at 12.5 μg to 2.9 cm at 100 μg, and for *C. neoformans*, the zones varied between 0.5 cm at 12.5 μg and 1 cm at 100 μg. These results indicated a dose-dependent antifungal activity of the extract, with higher concentrations leading to larger inhibition zones. However, despite the promising results, the extract showed lower inhibition, compared to itraconazole, a commercially available antifungal drug, which exhibited greater inhibitory effects even at lower concentrations (Figure 1, Table 1).

**Table 1:** Results of inhibition zone diameters (cm) from disc diffusion assay for *Aspergillus fumigatus*, *Candida albicans*, and *Cryptococcus neoformans*

Diameters of inhibition zones (cm) in disc diffusion assay (mean ± SD)			
*Concentration of Eisenia fetida* (mg/mL)	*Aspergillus fumigatus* (cm)	*Candida albicans* (cm)	*Cryptococcus neoformans* (cm)
100	1.7 ± 0.1	2.9 ± 0.2	1.0 ± 0.1
50	1.5 ± 0.1	2.7 ± 0.2	0.9 ± 0.1
25	1.2 ± 0.1	2.3 ± 0.1	1.0 ± 0.2
12.5	1.0 ± 0.1	2.2 ± 0.1	0.5 ± 0.1
Itraconazole	2.0 ± 0.1	3.5 ± 0.1	> 4.0

In the broth microdilution assay, the MIC and MFC values for the *E. fetida* extract were determined. The MIC/MFC values were 6.25%/12.5% for *A. fumigatus*, 1.56%/3.125%. for *C. albicans*, and 3.125%/25% for *C. neoformans*. These values suggest that the extract required higher concentrations to effectively inhibit fungal growth, especially compared to itraconazole, which showed superior efficacy at lower concentrations.

Although the extract demonstrated some antifungal activity against *A. fumigatus* and *C. albicans*, its efficacy was more pronounced against *C. albicans*, where it showed effective inhibition at relatively low concentrations. However, against *C. neoformans*, the extract displayed limited antifungal activity, requiring much higher concentrations to achieve both inhibitory and fungicidal effects. These findings suggest that while *E. fetida* extract possesses some potential as an antifungal agent, its effectiveness is species-dependent, highlighting the need for further studies to optimize its antifungal properties and enhance its therapeutic potential (Table 2).

**Table 2:** Minimum inhibitory concentration (MIC) and minimum fungicidal concentration (MFC) values of *Eisenia fetida* extract and itraconazole determined by broth microdilution assay

Fungal Species	Treatment	MIC (%) or (µg/mL)	MFC (%) or (µg/mL)
*Aspergillus fumigatus*	Itraconazole	0.5 µg/mL	1 µg/mL
	Eisenia fetida extract	12.5%	25%
*Candida albicans*	Itraconazole	0.25 µg/mL	0.5 µg/mL
	Eisenia fetida extract	3.125%	6.25%
*Cryptococcus neoformans*	Itraconazole	0.125 µg/mL	0.25 µg/mL
	Eisenia fetida extract	25%	50%

## Discussion

This study demonstrates the antifungal potential of *E. fetida* coelomic fluid against *C. albicans*, *A. fumigatus*, and *C. neoformans*. The extract showed the strongest inhibitory effects against *C. albicans* and *A. fumigatus*, with inhibition zones increasing in a dose-dependent manner, though consistently smaller than those of itraconazole. In broth microdilution assays, the extract had the lowest MIC and MFC for *C. albicans*, indicating potent inhibitory and fungicidal activity. *Aspergillus fumigatus* showed moderate sensitivity, requiring higher concentrations for fungicidal effects. These results align with those of previous findings indicating that filamentous fungi are generally more resistant to natural antifungal agents than yeasts [ 55 , 56 ]. Although the present study demonstrated promising antifungal activity of *E. fetida* extract, it is limited to *in vitro* assays and tested against a restricted panel of fungal species. Therefore, the findings may not fully translate into *in vivo* conditions.

Previous studies demonstrating the antimicrobial activity of earthworm extract have likely attributed to its diverse bioactive components, including antimicrobial peptides, enzymes, and lectins. Prior research has identified various earthworm-derived AMPs, such as Lumbricin-1, which exhibit broad-spectrum antimicrobial activity against bacterial and fungal pathogens [ 57 - 61 ]. Additionally, the inhibition of fungal growth may be linked to enzymatic degradation of fungal cell wall components, disruption of membrane integrity, or interference with key metabolic pathways [ 61 - 63 ]. Antioxidant compounds in earthworm coelomic fluid may also contribute to its antifungal properties by mitigating oxidative stress, a mechanism often exploited in fungal pathogenesis [ 40 , 64 - 66 ]. Wang *et al*. in their study demonstrated the antimicrobial effects of these compounds on *Escherichia coli* [ 59 ]. In another study, the antimicrobial peptide Lumbricin-1, which plays a role in the innate defense of the earthworm *Lumbricus rubellus*, was reported to show dose-dependent antimicrobial effects of the extract on *Porphyromonas gingivalis*, which aligns with the findings of the present study [ 67 ]. Other studies on the effects of earthworm extract on the growth and proliferation of microorganisms have revealed that the extract has strong antibacterial effects against *Shigella flexneri* and *Streptococcus pyogenes* [ 68 ]. It also broadly affects methicillin-resistant bacteria, including *Pseudomonas aeruginosa* and *Staphylococcus aureus* [ 69 ]. Zhoe *et al*. in their study examined the significant antifungal effect of earthworm extract on the fungus *Beauveria bassiana* [ 70 ]. In their study, it was found that the epidermal mucus of the earthworm *E. fetida* has a significant inhibitory effect on the extracellular fungal enzymes, disrupting fungal cell wall function and reproduction, ultimately inhibiting the fungus [ 71 - 74 ]. Generally, previous studies have focused on the effects of earthworm extract on bacteria and plant pathogenic fungi [ 75 , 76 ].

The present study highlighted the antifungal potential of *E. fetida* extract, particularly against *C. albicans* and *A. fumigatus*, supporting its further development as a natural antifungal agent. Future research should focus on *in vivo* efficacy, purification of active compounds, and elucidation of molecular mechanisms. Developing sustainable, safe, and cost-effective antifungal alternatives could offer a promising approach to complement or replace conventional treatments.

## Conclusion

This study demonstrated the *in vitro* antifungal potential of *E. fetida* coelomic fluid, suggesting its promise as a source of novel antifungal agents amid growing resistance to conventional therapies. These findings support its potential for clinical and agricultural applications. Further research is needed to isolate active compounds, clarify mechanisms of action, and evaluate their *in vivo* efficacy.
